# Supplementary material for: Dormancy‐to‐death transition in yeast spores occurs due to gradual loss of gene‐expressing ability
Source: Mol Syst Biol. 2020 Nov 18;16(11):e9245. doi: 10.15252/msb.20199245 (PMC7673291; doi:10.15252/msb.20199245)
Supplement: Supplementary file 2 — Movie EV1 [file MSB-16-e9245-s002.zip › Movie EV1.docx]

**Movie EV1 - RNAP II (Rpb3-mCherry) level in a spore bag that was aged for 39 days in water without nutrients and germinating 7 hours after receiving a 2%-glucose.**

A representative time-lapse movie of a spore bag that was incubated for 39 days in water without nutrients and then incubated for 24 hours in a minimal medium with a 2%-glucose (same data as in Fig. 5F). The 2%-glucose was added at the beginning of the movie. Each frame is spaced by 10 minutes. (Top left): Bright field channel, cropped field of view (7 μm x 7 μm). Red line represents the mask used for segmenting the spore bag. (Top right): mCherry fluorescence channel showing raw mCherry intensity inside the spore bag shown on top left. (Bottom): Average mCherry fluorescence inside the spore bag as a function of time. Black dot marks the moment of germination after 7 hours of being in the 2%-glucose.
