# Supplementary material for: Dormancy‐to‐death transition in yeast spores occurs due to gradual loss of gene‐expressing ability
Source: Mol Syst Biol. 2020 Nov 18;16(11):e9245. doi: 10.15252/msb.20199245 (PMC7673291; doi:10.15252/msb.20199245)
Supplement: Supplementary file 4 — Movie EV3 [file MSB-16-e9245-s004.zip › Movie EV3.docx]

**Movie EV3 - RNAP II (Rpb3-mCherry) level in a spore bag that was aged for 39 days in water without nutrients and germinating 12 hours after receiving a 2%-glucose.**

Same description as in Appendix Movie 1. Same data as in Fig. 5F. Black dot marks the moment of germination after 12 hours of being in the 2%-glucose.
